# Reports from the NICHD Global Network’s Maternal and Newborn Health Registry: supplement introduction

**DOI:** 10.1186/s12978-020-01024-4

**Published:** 2020-11-30

**Authors:** Robert L. Goldenberg, Shivaprasad S. Goudar, Sarah Saleem, Patricia L. Hibberd, Jorge E. Tolosa, Marion Koso-Thomas, Elizabeth M. McClure, for the Global Network investigators

**Affiliations:** 1 Department of Obstetrics and Gynecology, Columbia University, New York, NY USA; 2 Women’s and Children’s Health Research Unit, JN Medical College, KLE Academy of Higher Education and Research, Belagavi, Karnataka India; 3 Department of Community Health Sciences, Aga Khan University, Karachi, Pakistan; 4 Department of Global Health, Boston University School of Public Health, Boston, MA USA; 5 Oregon Health and Science University, Portland, OR USA; 6 Eunice Kennedy Shriver National Institute of Child Health and Human Development, Bethesda, MD USA; 7 Social Statistical and Environmental Health Sciences, RTI International, Durham, NC USA; 8 St. Luke’s University Health Network, Bethlehem, PA USA

In 2001, the National Institute of Child Health and Human Development of the National Institutes of Health (NIH) in the United States and the Bill & Melinda Gates Foundation funded the Global Network for Women’s and Children’s Health Research [1]. Its goal was and continues to be, to perform research to improve outcomes for pregnant women and their children in low-resource countries. The structure of the Global Network comprises partnerships between academic institutions in the United States and low and middle-income countries (LMIC) and a central data coordinating center, each funded by NIH grants which are recompeted periodically. Currently, the international sites are located in Pakistan, India (2 sites), Kenya, Zambia, the Democratic Republic of the Congo (DRC), Guatemala and Bangladesh. The Global Network has conducted more than 15 randomized clinical trials to assess the efficacy of various interventions to improve pregnancy outcomes and child health. In addition, since accurate data on pregnancy, maternal, fetal and neonatal outcomes and trends in these data over time are often not available in low-resource countries, the Global Network has also collected population-based data in specific geographic locations within each country. The Maternal and Newborn Health Registry, which began in 2008, now has collected data on more than 700,000 pregnant women and their outcomes. Research staff conduct visits to enroll women as early as possible during pregnancy, collect data during antenatal care, after delivery and at 42 days post-partum and perform multiple quality assurance checks to ensure the accuracy of Registry data with a focus on maternal, fetal and neonatal outcomes. To provide an overall picture of pregnancy and its outcomes and trends over time in LMIC, the Global Network developed this collection of papers. This supplement features 19 publications describing the Global Network and the Registry data from 2010 to 2018 [1–19].

This supplement covers a wide variety of topics related to pregnancy outcome and neonatal health, focusing on ways to improve outcomes for the pregnant women and their fetuses and newborns. The first several papers address the trends in the major outcomes, including maternal mortality [2], stillbirths [3], and neonatal mortality [4, 5]. The results presented in these papers suggest limited progress over the last decade. While maternal mortality is decreasing over time in each of the sites, we project that few, if any, will reach the goals established by the United Nations’ Sustainable Development Goals by 2030 [20]. The maternal mortality ratios in Pakistan and the DRC remain several times greater than the ratios in the other sites, and nearly 100 times greater than the ratios seen in the countries with the lowest maternal mortality ratios [2]. The stillbirth rates have also only declined modestly over the last decade [3] and across the Global Network, the stillbirth rate is greater than 20/1000 births, nearly tenfold higher than the rates in the countries with the lowest rates. Similarly, the neonatal mortality rates, while also declining slowly, are tenfold higher in the Global Network sites than in the countries with the lowest rates [4, 5].

The Global Network has invested heavily in the quality of data collected by improving the accuracy of assessment of gestational age and birthweight and their relation to outcomes. Saleem et al. explored neonatal mortality in babies born ≥ 2500 g [5]. Although most of the literature on neonatal outcomes has focused on adverse outcomes among preterm and low-birthweight infants, nearly half of the neonatal deaths in the Global Network occurred in neonates weighing ≥ 2500 g. Nearly all of these babies should survive with basic care. By comparison, Pusdekar et al. [6] and Marete et al. [7] address the relationship between mortality and preterm birth and low birthweight. Both studies indicate the need for new approaches to reducing neonatal mortality in these at-risk groups.

Several papers in the supplement explore how the location of delivery impacts fetal and neonatal mortality. With the global emphasis on transitioning to facility deliveries, Goudar et al. evaluated trends in facility deliveries in each Global Network site and how the increasing rates of facility delivery across the Global Network have impacted stillbirth and neonatal mortality rates [8]. In 2018, there was still wide variation in facility delivery rates across the sites, but pregnancy outcomes did not consistently improve with increasing facility delivery rates. The reasons for failure of facility-based care to improve outcomes in some sites is an important area for future research.

Mode of delivery was explored by Harrison et al., who addressed the increasing rates of cesarean delivery across the Global Network sites [9]. While cesarean delivery rates between 10 and 15% are thought to have the lowest maternal, fetal mortality and neonatal mortality rates [21], the Global Network African sites have cesarean delivery rates of less than 3%, which is clearly suboptimal. By contrast, Guatemala and the two sites in India have cesarean delivery rates greater than 20%, raising questions about overuse of this mode of delivery that does not appear to improve maternal, fetal or neonatal outcomes [22].

Unique trends in sites such as Pakistan, which has unacceptably high rates of maternal, fetal and neonatal mortality compared with the other Global Network sites have been observed [10]. Aziz et al. have reported on differences in population characteristics, medical care and pregnancy outcomes between the Pakistan site and the other Global Network sites and found a lower maternal literacy rate, a shorter inter-delivery interval and an increased risk of severe anemia. Rates of prematurity and low birthweight were higher in the Pakistan site and the data pointed to inadequate quality of care for the mothers and their fetuses and neonates. The Global Network data should be very useful for Pakistani policy makers and care providers to address quality of care for maternal and child health.

A series of papers explore the risks of adverse pregnancy outcomes associated with a variety of maternal, fetal and neonatal characteristics. These papers evaluate the associations of inter-delivery interval [11], parity [12], gender [13], anemia [14] and congenital anomalies [15] with pregnancy outcomes, including stillbirth and neonatal mortality. These papers confirm high rates of anemia in India and Pakistan, and especially the prevalence of severe anemia in Pakistan [14]. Short and long inter-pregnancy intervals were each associated with worse outcomes [11], male infants fared worse than female infants [13] and babies from the first and 4^th^ or greater pregnancies had worse outcomes than babies from the second and third pregnancies [12].

The Global Network has also been interested in the relationship between socioeconomic status and pregnancy outcomes. The paper by Patel et al. describes the creation of a new scale that the Global Network is using to determine the influence of socioeconomic status on outcomes [16]. This scale was used in several of the papers to provide additional insight into reasons for some of the poor outcomes across our sites.

Finally, the Global Network has put a great deal of effort into improving not only the quality of care for its subjects, but also into improving the quality of the data being collected. While metrics to assess quality have been a cornerstone of the Registry since its beginning, over time, improvements in data quality have occurred as reported by Garces et al. [17]. Patel et al. evaluated the quality of care around delivery related to early neonatal mortality [18]. In 2019, a new site in Bangladesh was added to the Global Network. Billah and colleagues describe the process to initiate a pregnancy outcome registry as well as their efforts to assure that accurate and reliable data are collected [19].

In summary, the Global Network Maternal Newborn Health Registry is among the largest and most accurate, ongoing prospective population-based pregnancy outcome databases in diverse geographical regions in LMICs. Importantly, the ongoing Registry, with its now more than a decade of historic data, will allow us to better understand the impact of new global outbreaks, such as the COVID-19 pandemic, on antenatal and obstetric care as well as major pregnancy outcomes. We anticipate that the Registry data will continue to provide insights into risk factors for adverse maternal, fetal, and neonatal outcomes in sites around the world, and should be useful for developing strategies to improve pregnancy outcomes in LMIC.

## Data Availability

Not applicable.
